# Conifer Regeneration After Experimental Shelterwood and Seed-Tree Treatments in Boreal Forests: Finding Silvicultural Alternatives

**DOI:** 10.3389/fpls.2018.01145

**Published:** 2018-08-17

**Authors:** Miguel Montoro Girona, Jean-Martin Lussier, Hubert Morin, Nelson Thiffault

**Affiliations:** ^1^Ecology Restoration Group, Department of Wildlife, Fish and Environmental Studies, Swedish University of Agricultural Sciences (SLU), Umeå, Sweden; ^2^Département des Sciences Fondamentales, Université du Québec à Chicoutimi, Saguenay, QC, Canada; ^3^Canadian Wood Fibre Centre, Natural Resources Canada, Ottawa, QC, Canada

**Keywords:** balsam fir, black spruce, ecosystem-based management, even-aged stands, partial cutting, seedling, shade-tolerant species, sustainable forest management

## Abstract

Forest regeneration is a key element in achieving sustainable forest management. Partial harvest methods have been used extensively in temperate broadleaf and mixedwood ecosystems to promote regeneration on poorly stocked sites and to maintain forest composition and productivity. However, their effectiveness in promoting conifer establishment has yet to be demonstrated in unmanaged boreal forests, especially those dominated by black spruce (*Picea mariana* (Mill.) BSP) where constraints for regeneration differ from those found in more meridional regions. We aimed to evaluate conifer seedling density and dimensions, 10 years after the onset of a gradient of silvicultural treatments varying in harvesting intensities, and to identify the critical factors driving the regeneration process. Study blocks of even-aged black spruce stands in the eastern Canadian boreal forest were submitted to three variants of shelterwood harvesting: a seed-tree harvest, a clear-cut and an untreated control. Shelterwood and seed-tree harvesting were combined with spot scarification to promote regeneration. Shelterwood and seed-tree harvesting produced a density of conifer regeneration sufficient to maintain forest productivity, but they did not promote seedling growth. Black spruce was the predominant species in terms of regeneration density, with proportions 3–5× higher than that for balsam fir (*Abies balsamea* (L.) Mill.). Ten years after treatment, seed-origin black spruce seedlings were abundant in skidding trails, while layers dominated the residual strips. Balsam fir density was not influenced by treatment nor by tree position relative to skidding trails. Balsam fir and black spruce had different responses to treatment in terms of height and diameter, the former exhibiting a better growth performance and larger diameter in the residual strips. Spot scarification created micro-sites that had a significant impact on the regeneration process. Overall, our results support that shelterwood and seed-tree harvesting combined with scarification enable adequate regeneration in black spruce stands, confirming these treatments as viable silvicultural alternatives to clear-cutting when required by sustainable forest management objectives.

## Introduction

Seedling establishment is of crucial importance to the persistence, productivity and resilience of forest ecosystems; adequate regeneration is critical in the sustainable management of the boreal biome (Prévost, 1996; Gauthier et al., 2009). While clear-cutting is the most widely-used harvesting method in North-America, it can lead to highly fragmented landscapes, declines in habitat diversity and losses of productivity (Groot et al., 2005; Fischer and Lindenmayer, 2007; Rosenvald and Lõhmus, 2008; Puettmann et al., 2015). Diversifying forestry practices and adapting silvicultural treatments are also necessary due to the pressures of climate change on forest ecosystems (Lindenmayer et al., 2012; Fedrowitz et al., 2014; Hof et al., 2017; Montoro Girona et al., 2018). The shelterwood and seed-tree systems are silvicultural alternatives to clear-cutting that can potentially address these concerns (Kern et al., 2017).

The shelterwood system favors the establishment and growth of regeneration through a uniform opening of the canopy, while limiting the growth of competing vegetation (Nyland, 2016). This system could be appropriate to ensure the regeneration of boreal conifers, maximize wood production and maintain biodiversity due to the high retention levels of forest stands (Vanha-Majamaa et al., 2007; Gauthier et al., 2009; Montoro Girona et al., 2017). Seed-tree harvesting, on the other hand, is a variant of clear-cutting that maintains only 5–30 seed-trees/ha, either in small groups or as dispersed individuals (Nyland, 2016). The remaining trees are chosen to provide sufficient seed sources after harvesting; the remaining cover is low, thus enabling light to reach the soil surface.

The shelterwood and seed-tree systems are potential options in current forest management strategies in Eastern Canada for two reasons: first, these systems may conciliate commercial harvest of timber and the maintenance of the key ecological attributes of mature stands, most important in ecosystem management strategies (Gauthier et al., 2009). Second, many jurisdictions rely on natural regeneration for the sustainable management of the boreal forest. Shelterwood or seed-tree systems can ensure and enhance regeneration, in particular in young and dense stands that often have marginal regeneration relative to current stocking standards (Oliver and Larson, 1996).

While the growth (Pamerleau-Couture et al., 2015; Montoro Girona et al., 2017) and mortality (Ruel et al., 2003; Anyomi and Ruel, 2015) of residual trees as well as the response of vegetation (Kneeshaw et al., 2002; Man et al., 2008) to partial harvesting have been documented for North American boreal forests, the factors contributing to the regeneration success remain unclear for most species under partial harvesting variants, including shelterwood and seed-tree cuts. In Eastern Canada, regeneration responses to shelterwood treatments have only been studied in silvicultural trials that lacked replication of treatments (Hatcher, 1961), were established in small, non-operational experimental designs (Raymond et al., 2000; Zarnovican et al., 2001) or were undertaken in mixedwood and deciduous stands (Tubbs, 1969; Metzger and Tubbs, 1971; Boivin, 1977). Moreover, whereas the regeneration of white spruce (*Picea glauca* [Moench] Voss) and balsam fir (*Abies balsamea* (L.) Mill.) has been documented in these contexts (Man and Lieffers, 1999; Beguin et al., 2009; Prévost and Gauthier, 2013), the establishment of black spruce (*Picea mariana* (Mill.) BSP) following partial harvesting and seed-tree cuts remains largely understudied (Kolabinski, 1991; Prévost, 1997) despite its ecological and economic importance (Giroud et al., 2016).

Ecological factors, such as soil characteristics and light availability, exert a marked influence on the establishment and growth of seedlings (Thiffault et al., 2015). Black spruce is a shade-tolerant species that mostly regenerates by layering from mature trees (>80%) in the absence of fire (Viereck and Johnston, 1990). However, seed-origin seedlings also contribute in maintaining productive stands (Lussier et al., 1992); and their establishment is highly dependent on the characteristics of the germination bed. For example, exposed mineral soil favors sexual regeneration success for this species (Jeglum, 1984; Prévost, 1997). Mechanical soil preparation through scarification following harvesting can improve seed-bed receptivity, as does wildfire under natural disturbance dynamics (Raymond et al., 2000; Zarnovican et al., 2001; Hille and Den Ouden, 2004). Removal of the forest canopy, even partially, affects light levels in the understory, with consequences on the availability of other resources (Canham et al., 1990; Lieffers et al., 1999; Coates, 2000; Raymond et al., 2006). However, no studies have yet documented the effects of the modified light regime following mechanized shelterwood or seed-tree harvesting on the regeneration success in black spruce–dominated stands.

Hence, we aimed to evaluate 10 years of regeneration for even-aged natural black spruce stands in the boreal forest of Eastern Canada that were subjected to three experimental variants of mechanized shelterwood, seed-tree and clear-cut silvicultural systems. Our working hypotheses were that (i) the combined effect of partial shading from residual trees and scarification allows an adequate regeneration density in the short- to mid-term for conifers, resulting in shelterwood variants and seed-tree methods that have a higher seedling density than clear-cutting when the former is combined with scarification; and (ii) seedling size is greater in seed-tree and clear-cut harvests relative to the shelterwood treatments, due to the high harvest intensity that increases light availability in the understory.

## Materials and methods

### Study area

**Figure 1:**
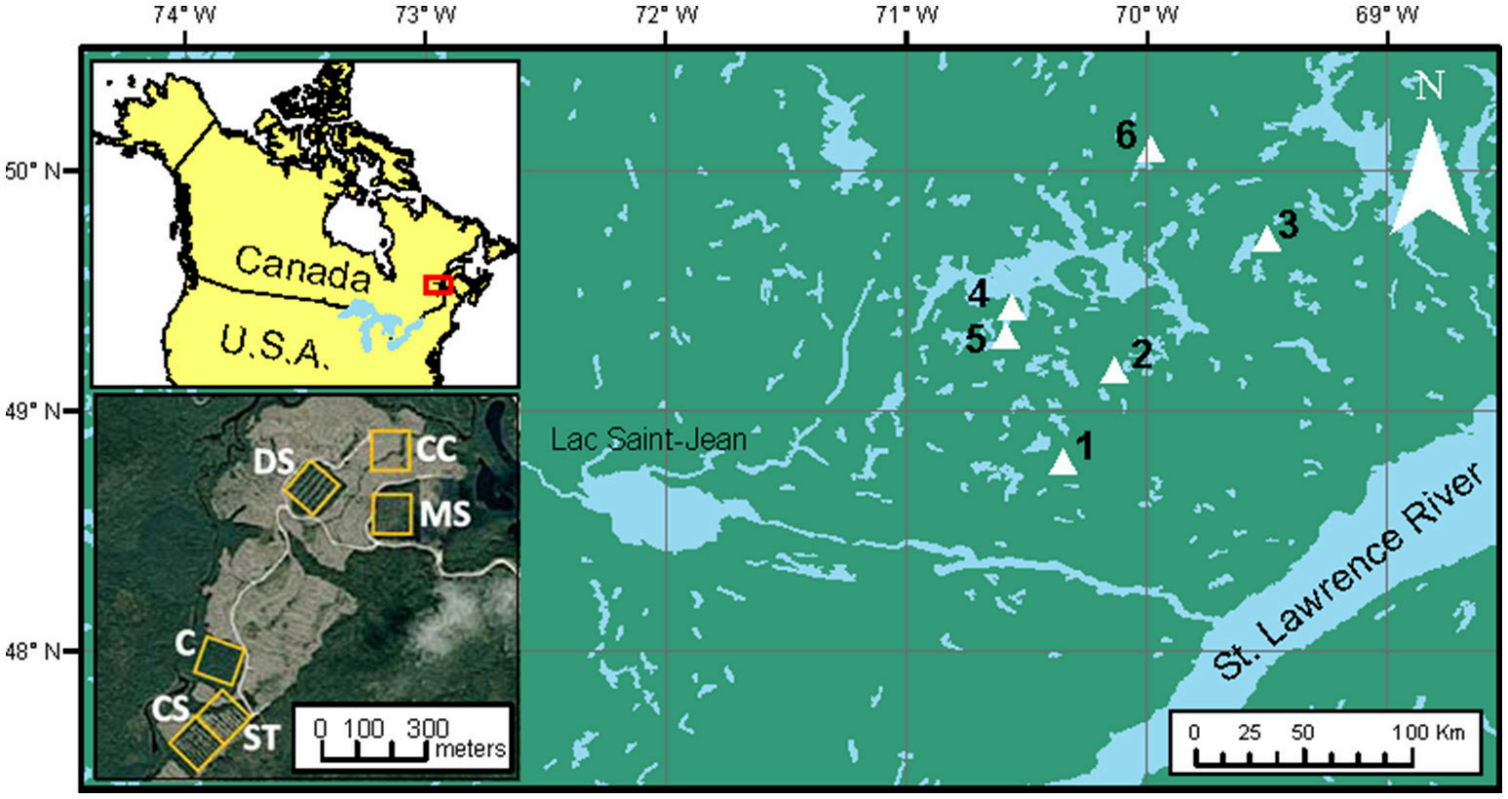
Location of the study area and experimental blocks (1–6). The orthophotography (lower-left corner) shows the 3-ha experimental units (yellow squares) of Block number 1 where letters refer to: (C), control; (MS), mini-strip; (DS), distant selection; (CS), close selection; (ST), seed-trees; and (CC), clear-cutting.

We conducted this study in even-aged, naturally established black spruce stands located in the Monts-Valin and North Shore regions of Quebec, Canada. The study areas lie within two bioclimatic regions, namely the balsam fir–white birch (*Betula papyrifera* Marsh.) and the black spruce–feathermosses bioclimatic domains (Saucier et al., 2009; Figure 1). The climate is subhumid subpolar, with a short vegetation season of 140 days (Rossi et al., 2011). Annual mean temperature ranges from −2 to 1.5°C and average annual precipitation ranges from 950 to 1,350 mm (Robitaille and Saucier, 1998). Surface deposits consist primarily of thick glacial tills, and rocky outcrops are frequent at the top of steep slopes (Robitaille and Saucier, 1998). The predominant soil type is humo-ferric podzols.

### Silvicultural treatments

**Figure 2:**
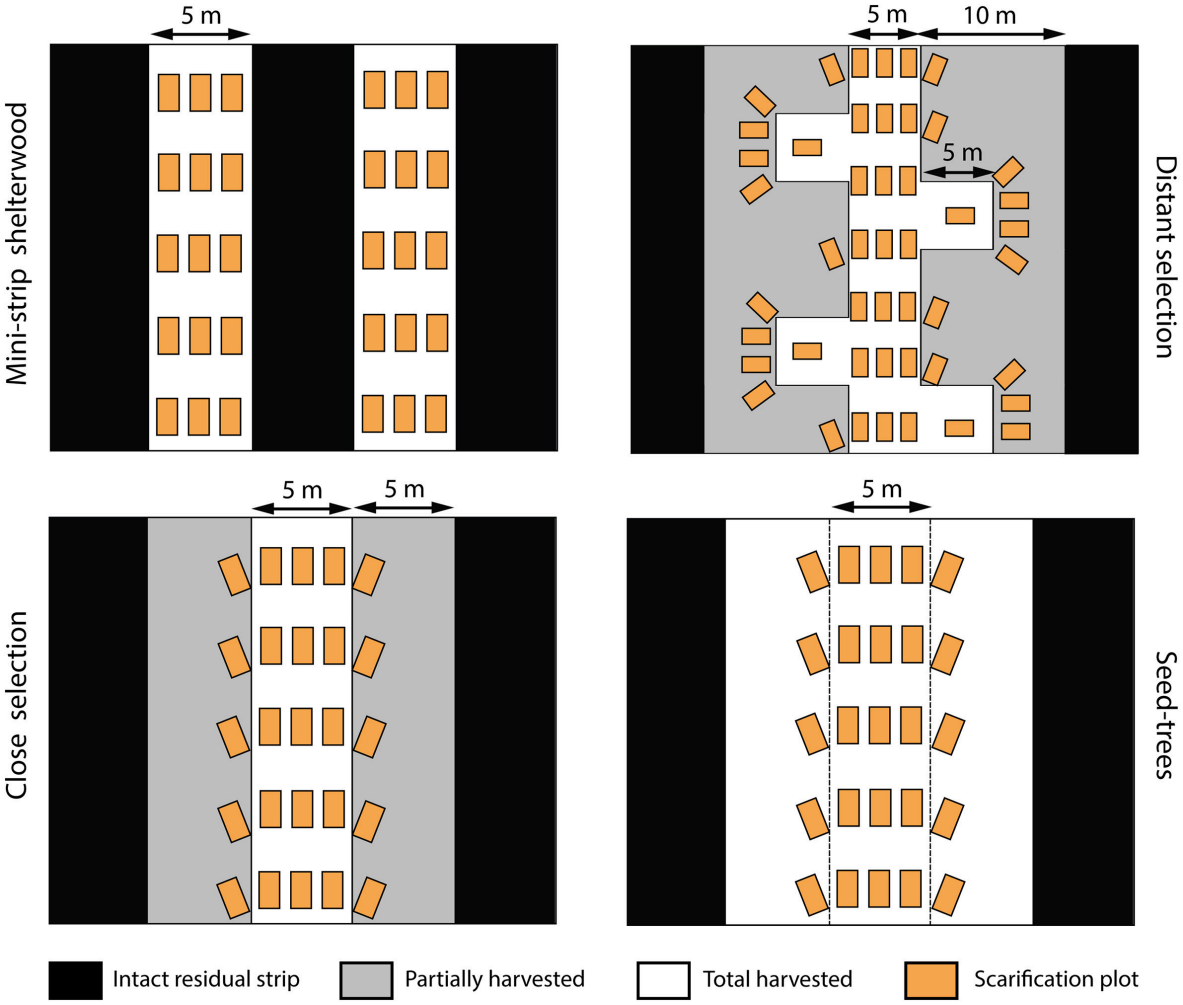
Spatial patterns of trails, residual strips and scarification plots in the study treatments. White areas represent the harvested surface or intervention trails, black areas indicate the intact residual strips, gray areas are the surface of the partially harvested residual strips and orange rectangles represent the scarification plots (2 m^2^). Scarification was not applied in the clear-cut and control plots.

Five harvesting treatments were performed in 2003 and compared in a fully replicated experimental design: mini-strip harvesting (MS), distant selection cutting (DS), close selection cutting (CS), seed-tree harvesting (ST), and clear-cutting (CC) with protection of advanced regeneration. The first three treatments are variants of a uniform shelterwood system (Montoro Girona et al., 2016). The main differences between the studied treatments were the spatial distribution of skidding trails and characteristics of the residual strips (Table 1). Harvest intensity, expressed in terms of proportion of basal area removed during the first harvest, was 50% for each shelterwood variant, 75% in ST and 100% for CC. MS consisted of a succession of 5-m-wide cut strips, with 5-m-wide residual strips. ST had wider cut strips (15 m) than MS, with 5-m-wide intact residual strips. In the case of CS and DS, trails were established at 20- and 30-m intervals, respectively, and the stand was partially harvested on each side of the trails, at a maximum distance of 5 m from the trail edge. DS had short secondary trails, perpendicular to the main operational trails. Each was separated by 10 m. In 2004, we designed and applied various patterns of soil scarification in each treatment (except in CC) within 2 m^2^ rectangular plots (Figure 2). Scarification was performed using a 10-ton excavator equipped with a 1 m^3^ bucket.

**Table 1:** Characteristics of the silvicultural treatments.

**Treatment**	**Basal area harvested (%)**	**Residual strip**		**Skidding trail**			**Secondary trail**	**Scarification density (m**^**2**^**/ha)**	
		**Width (m)**	**Intact surface (%)**	**Width (m)**	**Area (%)**	**Spacing (m)**		**Prescribed**	**Obtained^b^**
Control	0	–	–	–	–	–	–	0	0
Mini-strip (MS)	50	5	100	5	50	5	No	1,500	1,321 (256)
Close selection (CS)	50	15	33	5	25	20	No	1,250	752 (274)
Distant selection (DS)	50	25	20	5 or 10^a^	17	30	Yes	1,050	538 (144)
Seed-trees (ST)	75	5	100	15	75	20	No	1,250	844 (258)
Clear-cut (CC)	100	0	0	0	0	0	No	0	0

a *Corresponds to the variability in the intervention as a consequence of secondary trails*.

b *Standard error is shown in parentheses*.

### Experimental design

**Figure 3:**
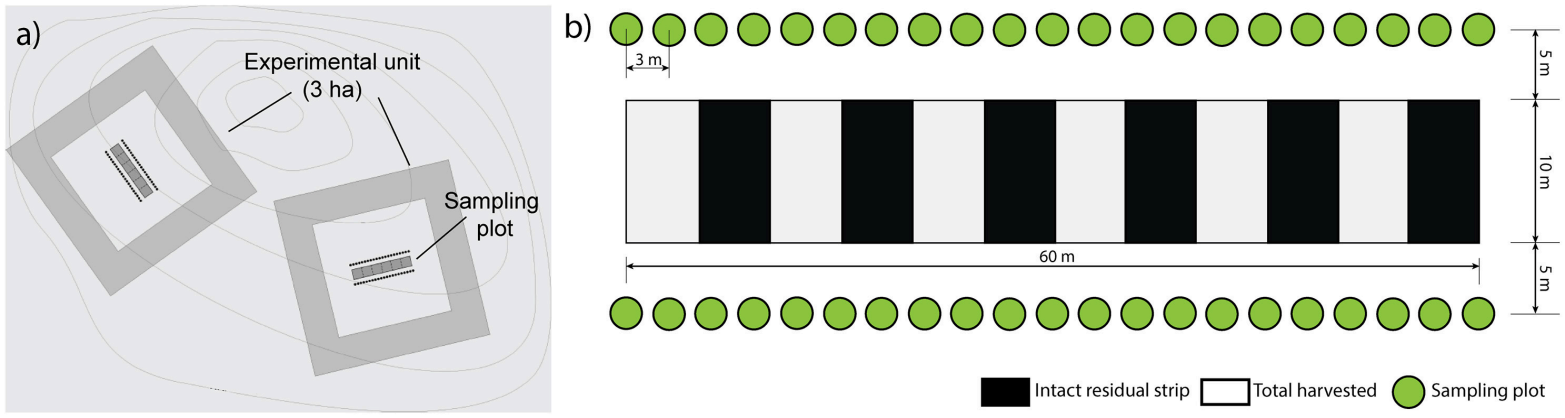
Sampling design. **(a)** Location of plots within the experimental units. **(b)** Distribution of the regeneration micro-plots (4 m^2^).

We set up the experiment as a factorial design with completely randomized blocks. Six blocks were sampled, each one including six experimental units of 3 ha each, corresponding to one replicate of each silvicultural treatment and one untreated control plot (Figure 2). Two stand types were selected: three blocks were established in dense, relatively young forests (80–100 years, average density of 2,600 trees/ha), characterized by a low level of pre-established regeneration (average density of 2,600 trees/ha), and three blocks in open and relatively old forests (120–150 years), characterized by a high level of pre-established regenerated (average density of 1,500 trees/ha). In all cases, black spruce was the dominant species, accounting for at least 90% of the stand basal area (Table S1). Within each block, we situated the experimental units in areas that were relatively homogeneous in terms of species composition and stand density. We installed a permanent rectangular (10 × 60 m) sampling plot in the center of each experimental unit, perpendicular to the main skidding trails (Figure 3a). Two transects, each comprising 21 circular micro-plots (4 m^2^), were established parallel to the sampling plots to study the regeneration response to the treatments (42 micro-plots × 6 treatments × 6 blocks = 1512 micro-plots). The sampling design was such that it covered the within-stand spatial heterogeneity of each harvesting treatment (Figure 3b). Overall, the experimental factors included the combinations of stand types (younger, older stands) and the silvicultural treatments (MS, DS, CS, ST, CC, Control).

### Regeneration assessment

Measurements were taken 1 year before cutting (b.c.) as well as 1 and 10 years after cutting (a.c.). We performed two inventories to study seedling density and growth. First, all seedlings in each micro-plot were counted and classified by species and height class (0–4.9, 5–29.9, 30–99 cm, and >1 m). Second, in each micro-plot, we selected one dominant seedling to evaluate the regeneration response 10 years after treatment. For each selected individual, we noted height, diameter, age (whorl count), origin (sexual or vegetative), rooting substrate (woody debris, mineral soil, dead wood and vegetation cover by stratum) and moss species found at the base. Other ancillary data were also collected at the seedling level: micro-plot disturbance as the percentage of soil surface affected (in four classes: 0–25% undisturbed, 25–50% moderate, 50–75% high, and 75–100% very high) and the type of disturbance (rut, mound, scarification, windthrow or intact forest floor). The spatial position of micro-plots relative to the harvesting trails was also noted (strip, edge, and trail). Edge surface was considered as the area within 1.25 m on each side of the trails. We calculated seedling mortality due to the treatment as the difference in the number of seedlings b.c. and 1 year a.c. at the micro-plot level. Solar radiation was measured b.c. and 10 years a.c. as the percentage transmittance of photosynthetically active radiation (PAR; 400–700 nm) by leaving a quantum sensor and a data logger at 1-m above the ground, while another sensor was positioned in fully open conditions to measure the incident PAR as a control (Lieffers et al., 1999; Paquette et al., 2007). Two measurements of solar radiation were performed and averaged for each micro-plot (one parallel and one orthogonal to micro-plot orientation).

### Statistical analyses

We conducted an analysis of variance (ANOVA) to evaluate treatment effects on the density of seedling regeneration by species, 10 years a.c. using the MIXED procedure of SAS 9.2 (SAS Institute, Inc., Cary, NC, USA). The model included blocks as a random effect, and stand type, treatment and their pairwise interactions as fixed effects. Natural logarithmic transformation of density values was used to satisfy the assumptions of normality and homogeneity of variance. We used the SLICE statement of the MIXED procedure to partition analyses of the LS-means in the case of significant interactions (*p* < 0.05). The same model was used to evaluate treatment effects on stocking and seedling size variables (height, diameter and age). Stocking was defined as the proportion of regeneration plots with a least one living seedling. The observed stocking was compared with the expected stocking assuming a random dispersion of seedlings in the stand. We assumed that the distribution frequency of the number of seedling per 4 m^2^ plot followed a Poisson distribution.

As such, the expected probability of having at least one seedling per plot, considering the average density of seedlings, was equal to:

$$\begin{array}{l} P\left(n>0\right)=1-e^{-average\;density} \end{array}$$

Principal component analyses (PCA) were conducted to elucidate the relationships between the most influential factors on regeneration density and size, for both black spruce and balsam fir. We ran PCA with the FACTOR procedure of SAS 9.2 (SAS Institute, Inc., Cary, NC, USA) using all the variables collected at the micro-plot level.

## Results

### Density, stocking, age and size of seedlings

**Figure 4:**
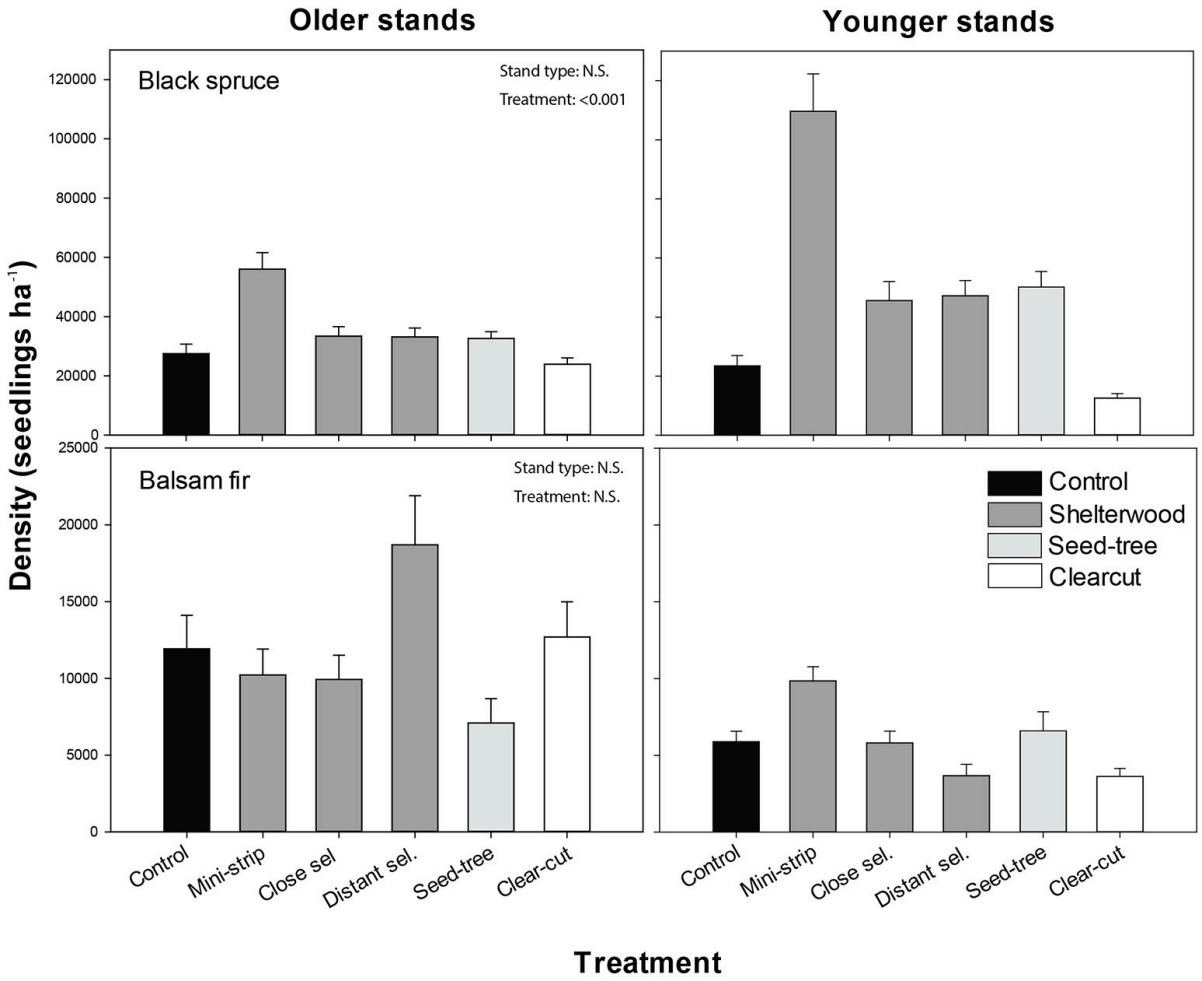
Density of regenerating seedlings, 10 years after treatment for each combination of stand type and species. Vertical bars show the standard error. Contrasts are shown in Table 3.

Black spruce was the dominant regenerating species a.c., with seedling densities that were three to five times higher than that of balsam fir, 1 and 10 years a.c., respectively. Black spruce regeneration density 10 years a.c. was significantly affected by treatments in interaction with stand types (Figure 4). Younger stands exhibited seedling densities that were at least 3 times higher a.c. compared to b.c., whereas older stands showed only slightly higher values 10 years a.c. than b.c. (Table 2). In both stand types, black spruce seedling density increased significantly in the scarified shelterwood and ST plots compared to control and clear-cut plots (Figure 4; Table 3). MS was the most effective treatment to promote black spruce regeneration in both stand types. The treatment × stand type interaction was caused by significant differences in CC. In younger stands, CC showed the lowest density of seedlings 10 years a.c. (even with the control), while in older stands, the values were similar to the other studied treatments, with the exception of MS (Figure 4). For balsam fir, stand type and treatments did not significantly influence seedling density (Table 3; Figure 4). However, some trends were observed, as balsam fir density was two times higher in older stands than in younger stands a.c. Shelterwood and ST followed by scarification induced 35% mortality of pre-established regeneration at 1-year a.c.

**Table 2:** Stocking and seedling density of conifer regeneration before cutting (mean ± standard error).

**Stand type**	**Number of micro-plots**	**Black spruce stand proportion (%)**	**Stocking (%)**		**Seedling density (seedlings ha**^**−1**^**)**	
			**Black spruce**	**Balsam fir**	**Black spruce**	**Balsam fir**
Older	756	95.6 ± 2.8	83.5 ± 2.6	37.6 ± 6.0	30, 998.7 ± 446.7	6, 835.3 ± 775.1
Younger	756	96.1 ± 2.3	51.9 ± 7.2	21.0 ± 7.48	9, 990.1 ± 294.8	1, 362.4 ± 505.3

**Table 3:** Analysis of variance (ANOVA) results for seedling density 10 years after cutting, by seedling species.

			**Black spruce**		**Balsam fir**	
**Effect**	**df**	**ddf**	***F***	***Pr > F***	***F***	***Pr > F***
Stand type	1	4.0	0.03	0.88	4.13	0.12
Treatment	5	20.2	27.90	<**0.001**	0.45	0.81
Control vs. Treated	1	20.2	−4.54	**0.001**	–	–
(MS-CS-DS) vs. (ST-CC)	1	20.2	6.43	<**0.001**	–	–
MS vs. (CS-DS)	1	20.2	4.89	**0.001**	–	–
CS vs. DS	1	20.2	−2.20	**0.04**	–	–
ST vs. CC	1	20.2	7.03	<**0.001**	–	–
Stand type × Treatment	5	20.2	5.15	**0.003**	2.24	0.11
(Control vs. Treated) × (Older vs. Younger)	1	16.2	0.24	0.82	–	–
[(MS-CS-DS) vs. (ST-CC)] × (Older vs. Younger)	1	16.2	−1.87	0.08	–	–
[MS vs. (CS-DS)] × (Older vs. Younger)	1	16.2	−0.36	0.72	–	–
(CS vs. DS) × (Older vs. Younger)	1	16.2	0.51	0.62	–	–
(ST vs. CC) × (Older vs. Younger)	1	16.2	−3.51	**0.002**	–	–
Stand type × Treatment (SLICE)						
Control (Older vs. Younger)	1	8.7	0.68	0.43	–	–
MS (Older vs. Younger)	1	7.0	1.53	0.26	–	–
CS (Older vs. Younger)	1	7.0	0.03	0.86	–	–
SD (Older vs. Younger)	1	7.5	0.34	0.58	–	–
ST (Older vs. Younger)	1	7.0	0.17	0.70	–	–
CC (Older vs. Younger)	1	7.2	5.68	**0.05**	–	–

*The analysis assumed a mixed model in which the fixed effects were the two stand types and the five cutting treatments plus a control. MS, mini-strip; CS, close selection; DS, distant selection; ST, seed-tree and CC, clear-cutting. Only orthogonal contrasts are shown. Significant values are shown in bold (p < 0.05)*.

Initial black spruce stocking values were 52 and 84% for younger and older stands, respectively, with lower values for balsam fir (21 and 38%, respectively, Table S2). Stocking, 10 years after the shelterwood and ST treatments combined with scarification, ranged from 71 to 94%. In younger stands, black spruce stocking a.c. was, in most cases, 40% higher than b.c., whereas in older stands, stocking was 10% higher a.c. than b.c. For balsam fir, we observed similar stocking levels a.c. and b.c. In all cases, the observed stocking was lower than the expected stocking. Hardwood stocking 10 years a.c. was 10% higher than b.c. levels for both stand types (Table S1). CC and ST had the highest stocking values, at 56 and 71%, respectively, while the shelterwood variants with scarification showed lower values (43–56%).

**Figure 5:**
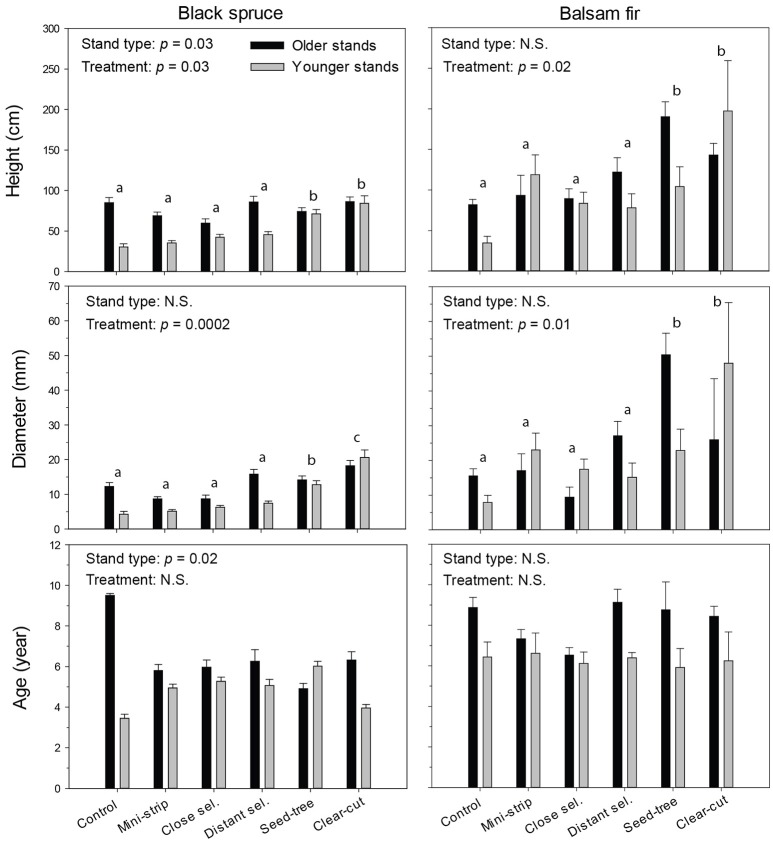
Dimensions and age of regenerating seedlings, 10 years after treatment for each combination of stand type and species. Vertical bars show the standard error. Bars with the same letters are not significantly different at α = 0.05.

The growth characteristics of seedlings showed that balsam fir and black spruce had different responses to treatments in terms of height and diameter, 10 years a.c. (Figure 5). In that regard, both species were influenced by the treatment effect; although in the case of black spruce, regeneration significantly responded to stand type as well (Tables 4, 5). For black spruce, mean height ranged from 30 to 86 cm and diameter varied from 4 to 21 mm. Black spruce had higher values for height and diameter (1.4–2.8× higher) in older stands than in younger stands for shelterwoods with scarification and control plots, while responses to stand types were similar in CC and ST with scarification (Table 4). Age of black spruce seedlings ranged from 4 to 6 years, with no difference among treatments (*p* = 0.85). However, seedling age was significantly higher in older stands than in younger stands (*p* < 0.02). Balsam fir seedlings ranged from 34 to 197 cm in height and 9–50 mm in diameter (Figure 5). Treatments had a significant effect on balsam fir height and diameter, with higher values for treated plots than the controls; however, we detected no significant differences between stand types and their interaction (Table 5). Seedling age was higher for balsam fir (from 6 to 9 years), and it was similar between stand types and treatments, although being slightly higher in older stands. We detected no significant relationship between available insolation and seedling density or size for both species (Figure S1).

**Table 4:** Analysis of variance (ANOVA) results for black spruce seedling characteristics.

			**Height**		**Diameter**		**Age**	
**Effect**	**df**	**ddf**	***F***	***Pr > F***	***F***	***Pr > F***	***F***	***Pr > F***
Stand type	1	4	10.16	**0.03**	2.87	0.17	9.41	**0.02**
Treatment	5	20	3.20	**0.03**	8.41	<**0.001**	0.39	0.85
Control vs. Treated	1	20	0.85	0.41	1.58	0.13	–	–
(MS-CS-DS) vs. (ST-CC)	1	20	−3.35	**0.003**	−5.22	<**0.001**	–	–
MS vs. (CS-DS)	1	20	0.69	0.50	1.32	0.20	–	–
CS vs. DS	1	20	1.46	0.16	1.88	0.07	–	–
ST vs. CC	1	20	1.21	0.24	−2.66	**0.02**	–	–
Treatment × Stand type	5	20	1.88	0.14	1.58	0.21	2.95	**0.04**
(Control vs. Treated) × (Older vs. Younger)	1	20	–	–	–	–	0.82	0.42
[(MS-CS-DS) vs. (ST-CC)] × [Older vs. Younger]	1	20	–	–	–	–	0.89	0.39
[MS vs. (CS-DS) vs. (ST-CC)] × [Older vs. Younger]	1	20	–	–	–	–	0.71	0.49
[CS vs. DS] vs. [Older vs. Younger]	1	20	–	–	–	–	0.54	0.60
[ST vs. CC] × [Older vs. Younger]	1	20	–	–	–	–	2.64	**0.02**

*The analysis assumed a mixed model in which the fixed effects were the two stand types, five cutting treatments plus a control and their interaction. MS, mini-strip; CS, close selection; DS, distant selection; ST, seed-tree; and CC, clear-cutting. Only significant orthogonal contrasts are shown. Significant values are shown in bold (p < 0.05)*.

**Table 5:** Analysis of variance (ANOVA) results for the seedling characteristics of balsam fir.

			**Height**		**Diameter**		**Age**	
**Effect**	**df**	**ddf**	***F***	***Pr > F***	***F***	***Pr > F***	***F***	***Pr > F***
Stand type	1	2	0.42	0.58	0.13	0.75	12.88	0.11
Treatment	5	8	4.82	**0.02**	7.81	**0.01**	1.00	0.46
Control vs. Treated	1	8	−3.56	**0.02**	−3.71	**0.01**	–	**–**
(MS-CS-DS) vs. (ST-CC)	1	8	−2.95	**0.01**	−4.46	**0.001**	–	**–**
MS vs. (CS-DS)	1	8	0.75	0.48	0.19	0.86	–	**–**
CS vs. DS	1	8	1.79	0.11	−2.44	**0.04**	–	**–**
ST vs. CC	1	8	0.30	0.77	0.08	0.93	–	**–**
Treatment × Stand type	5	8	1.80	0.22	2.91	0.08	0.98	0.47

*The analysis assumed a mixed model in which the fixed effects are the two stand types, five cutting treatments plus a control and its interaction. MS, mini-strip; CS, close selection; DS, distant selection; ST, seed-tree; and CC, clear-cutting. Only significant orthogonal contrasts are shown. Significant values are shown in bold (p < 0.05)*.

Regeneration distribution in the various height classes differed among stand types, treatments and species, and in most cases showed unimodal distributions (Figure S2). The height of black spruce seedlings was less variable among stand types and treatments than the height of balsam fir seedlings. Most black spruce seedlings 10 years a.c. were found in the 5–29 cm height class, with the exception of CC and ST, in which regeneration seedlings were taller (30–99 cm). Overall, seedlings were smaller in shelterwood variants than in the control. The height distribution of balsam fir differed, especially in older stands, where we observed two dominant height classes (5–29 cm; 30–99 cm). Ten years after the experimental shelterwood cutting and for both stand types, most of balsam fir seedlings were between 5 and 29 cm (as in the control plots). However, DS had a greater variability in height and taller seedlings in older stands than in younger stands. For CC (in both stand types) and ST (in older stands only), the majority of balsam fir seedlings were taller than those under shelterwood and control conditions, ranging from 30 to 99 cm in height.

### Multivariate analyses of the regeneration process

Black spruce accounted for 85% of the measured seedlings. In shelterwoods and ST (having scarification after the treatment), seedlings originated mostly from sexual reproduction, although differences were detected between younger (proportion of sexual/asexual: 51–76%) and older stands (29–63%). Layering was the main origin of seedlings in the control and CC plots, accounting for 88–99% and 66–86% of regeneration, respectively. Seedlings originating from sexual reproduction were mainly located in trails, whereas vegetative layers were mostly found in the residual strips. *Polytrichium* sp. was the dominant moss species observed in trails, while residual strips were mostly covered by *Pleurozium* sp. and *Hylocomium* sp. Trails were characterized by a high disturbance level; most of their surface (50–100%) was dominated by mineral soil exposed from the scarification treatments, as well as mounds, ruts and woody debris. The soil in the residual strips was mostly undisturbed (intact forest floor).

**Figure 6:**
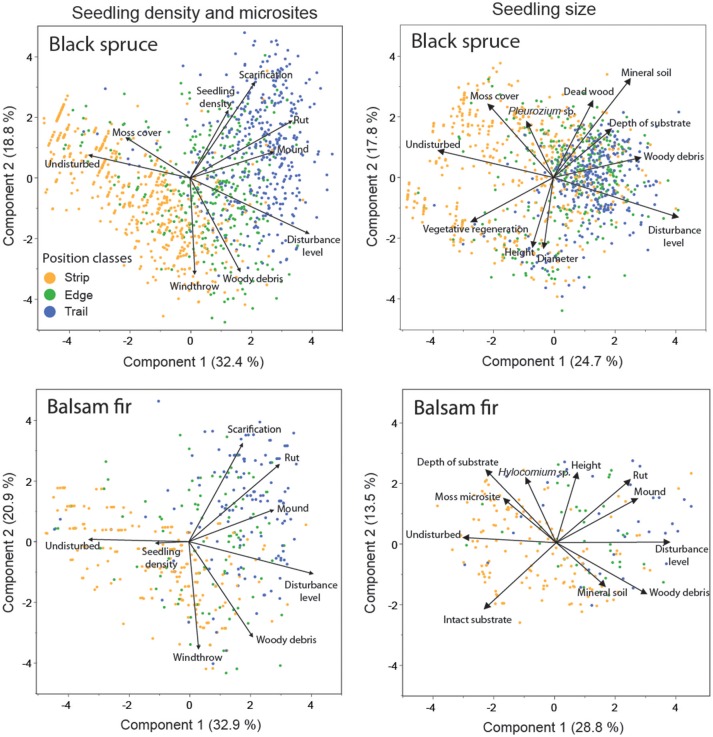
Principal component analysis of micro-plot variables, seedling density and seedling size for black spruce and balsam fir, respectively. Each point represents a micro-plot. The proportion of the explained variance is indicated for each axis.

PCA showed that position relative to the trails influenced the regeneration process for both black spruce and balsam fir. The analyses identified a common group of micro-plot factors related to either sexual seedlings on trails (rounds, mounds and high disturbance level), edges (windthrow and woody matter) or vegetative saplings on residual strips (moss cover and undisturbed micro-plots) (Figure 6).

For black spruce regeneration density, the first two axes of the PCA explained 51% of the variance (Figure 6, top-left). Scarification, position and ruts correlated strongly with the regeneration density of this species. PCA revealed that black spruce seedling density was highly correlated with scarification. The first two axes of the PCA explained 54% of balsam fir regeneration density. However, correlations were lower than for black spruce, as indicated by a short vector for this variable on the first axis and no correlation with the second axis (Figure 6, bottom-left). Balsam fir regeneration was mainly located on intact residual strips.

PCA, using seedling height, diameter and micro-plot characteristics, explained about 43% of the variation for black spruce and balsam fir seedlings and revealed differences between species (Figure 6, top-right and bottom-right, respectively). Black spruce seedlings were separated into two groups, one associated with a vegetative origin in residual strips in older stands, the other associated with a regeneration having a sexual origin in the trails of younger stands. Vegetative regeneration was associated with tall seedlings, large diameters and undisturbed seed-beds covered with moss. Sexual regeneration was associated with harvest trails having an exposed mineral soil and *Pleurozium* sp. Most pre-established balsam fir seedlings were in the residual strips, characterized by undisturbed micro-plots, although post-cutting seedlings were also identified in the trails, especially on mounds having a high amount of woody debris.

## Discussion

Understanding and quantifying regeneration responses after harvesting in general, and partial cutting in particular, is essential to identify optimal forest management strategies that can support ecosystem-based management objectives (Messier et al., 1999). Our study confirms, for the first time, that experimental shelterwood and seed-tree harvesting followed by scarification allow the establishment of an abundant black spruce regeneration in North American boreal forests. In combination with soil scarification, maintaining residual trees in shelterwoods and seed-tree treatments improved regeneration density and stocking compared to control plots, particularly in younger and denser stands. Mini-strip harvesting followed by scarification resulted in the highest seedling density among the treatments, in conjunction with the highest density of scarified micro-plots (a result of the highest proportion of skidding trails). However, major differences were observed between the prescribed and the obtained density of scarified spots, the resulting density being 12–49% lower than expected. Residual trees and dense patches of advanced regeneration prevented the creation of more scarified micro-sites. Control plots and clear-cuts were not scarified and had the lowest level of black spruce regeneration densities; values were in line with those observed in previous studies conducted in boreal ecosystems (Harvey and Brais, 2002; MacDonald and Thompson, 2003). Clear-cutting induced little damage to advanced regeneration, as it was performed following careful logging practices, limiting soil disturbance, which in turn restricted regeneration establishment (Greene et al., 1999). The lack of soil disturbance is not the only factor that can explain the low densities found in clear-cut plots. The limited range of seed dispersal also played a role. Prévost (1997) observed that stocking decreases linearly with distance from seed-tree groups, up to 50 m in adjacent stands (2.5–3× farther than stand height). Thus, shelterwood variants and seed-tree harvesting combined with scarification were likely more effective than clear-cutting in promoting sexual regeneration because of the reduced distance from seed sources, increased soil humidity, decreased maximum air temperature and minimized frost occurrences and severity (Man and Lieffers, 1999; MacDonald and Thompson, 2003). Soil scarification may have similar effects in clear-cuts than in alternative treatments, but only in a limited distance from seed trees from the uncut forest border. The treatments have been designed to promote natural regeneration. As seed dispersion in black spruce stands depends on the distance of residual forests, seedling establishment in clear-cuts will be dependent on harvesting block size. Otherwise, we would expect only marginal establishment of regeneration in this case. We also hypothesize that spot scarification reduced the direct competition for light by feathermosses, which have a similar height to black spruce germinants.

Balsam fir characterized the secondary regeneration species, 10 years after partial cutting, although the species was more abundant than in the original stand (which was >95% black spruce). This is an important issue as after silvicultural intervention in black spruce stands, species composition can be altered and balsam fir can become the dominant species in these stands. This phenomenon increases the vulnerability of stands to spruce budworm, the most important defoliator in eastern Canadian forests (Maclean, 1996; Montoro Girona et al., 2018). Based on our results, this change in species composition after treatment was not enough to significantly increase stand vulnerability. Density of balsam fir was not influenced by stand type, treatment nor spatial position. This suggests that the pre-treatment light and soil conditions may not have been factors limiting the establishment of balsam fir, but rather it was the density and distribution of seed-trees in these spruce-dominated stands. However, balsam fir density after CC in older stands was similar to that reported by Harvey and Brais (2002), whereas in younger stands, density was two times lower. Shelterwood and ST systems modified the light regime in both space and time (by harvesting operations and the windthrow that followed), but it did not favor the establishment of shade intolerant competitors (i.e., deciduous species).

The growth of regenerated black spruce is slow in comparison with other conifer species, such as jack pine (*Pinus banksiana* Lamb.), tamarack [*Larix laricina* (Du Roi) K. Koch] (Thiffault et al., 2004), and balsam fir (Doucet and Boily, 1995; Messier et al., 1999). Ten years after treatment, black spruce height was similar to that reported in other studies (Harvey and Brais, 2002; Thiffault et al., 2004; Renard et al., 2016). Mean annual growth was 8.3 cm·yr^−1^ in shelterwoods and 9.5 cm·yr^−1^ in CC and ST. These growth rates were higher than those observed by Harvey and Brais (2002) (6.1 cm·yr^−1^), but lower than the 15 cm·yr^−1^ reported by Boily and Doucet (1993), 7–8 years a.c. The dominant seedling height classes 10 years a.c. corresponded to those that Riopel et al. (2011) observed at 5 years a.c. This was likely due to the stand not being even-aged, and the regeneration being mainly composed of pre-established layers in the latter study. CC and ST followed by scarification showed the best growth performance for black spruce and balsam fir. This is in agreement with MacDonald and Thompson (2003) who noted that the height and diameter of planted conifers increased with harvest intensity in a boreal mixedwood forest. We observed that shelterwood with scarification was not the most efficient treatment to promote seedling growth 10 years a.c. Growth performance increased with harvest intensity; differences were more than 2 cm·yr^−1^ between ST-CC and experimental shelterwoods.

For many species, natural regeneration by seed depends on the receptivity of germination beds (Galipeau et al., 1997; Hille and Den Ouden, 2004). Even-aged black spruce stands are derived mostly from seeds that germinate after fire (Greene et al., 1999; Gagnon and Morin, 2001), whereas advanced regeneration is largely dominated by layers. Our results show that all treatments that removed the soil organic layer produced a higher seedling density than those maintaining a high proportion of undisturbed forest floor (Hille and Den Ouden (2004). Scarification, in combination with micro-plot position relative to the intact strips and trails, favored black spruce germination by exposing the mineral soil and providing lateral shadow from residual strips (Messier et al., 1999). Black spruce regeneration was mostly concentrated in the trails, where the removal of the organic layer likely increased water availability and decreased early competition from moss. Our results showed that scarification was essential for achieving the satisfactory establishment of black spruce regeneration (Prévost (1996), an effect also observed with other species (Nilsson et al., 2002; Hille and Den Ouden, 2004).

Spatial position played an important role in the distribution of species and the type of regeneration. Black spruce seedlings located in the scarified trails were mostly of sexual origin, while those found in the residual strips were mostly of asexual layering. Trails had a black spruce density six times greater than for residual strips without any soil preparation, a result matching observations in white spruce stands (Solarik et al. (2010). Without proper site preparation, regeneration in trails can be lower than in residual strips (Riopel et al., 2011). Balsam fir, on the other hand, has larger seeds than black spruce and can successfully grow roots and survive in undisturbed humus layers (Greene and Johnson, 1998). Hence, balsam fir regeneration was mostly located in the residual strips (Harvey and Brais, 2002; Riopel et al., 2011). Black spruce and balsam fir also have different regeneration responses in terms of size and density (Figure 5). Under shade conditions, suppressed balsam fir modify their crown architecture and favor a lateral expansion at the expense of vertical growth, hence producing high survival rates in residual strips (Messier et al., 1999).

Growth and survival of seedlings under shady conditions involves the complex interaction between the plant and resources, such as light, nutrients and water availability (Messier et al., 1999). Several studies have demonstrated than black spruce layers surviving in the understory for more than 100 years is common (Morin and Gagnon, 1991), and that the age of balsam fir saplings in the understory has been substantially underestimated as this species can have up to 40 missing rings (Morin and Laprise, 1997). Consequently, residual strip regeneration was the most challenging to correlate with environmental changes induced by silvicultural treatments for both conifer species (Figure 6).

Black spruce and balsam fir regeneration was not related to insolation levels during the first 10 years a.c. This can be explained by the intermediate levels of harvest intensity applied in our study, levels not severe enough to promote high light levels. However, we expect that light availability, as influenced by competing vegetation (i.e., deciduous species) will influence seedling growth and survival in the coming years. Long-term monitoring will be necessary to verify the impacts of insolation and deciduous species' competition on seedling growth.

Organic matter, sphagnum moss and mineral soil are the ideal seed-beds for establishing black spruce as they promote high seedling survival and density (Zasada et al., 1992; Duchesne and Sirois, 1995; Prévost, 1997; Raymond et al., 2000). Black spruce seedlings were mostly found in concave micro-sites created by scarification treatments, similar to the observations of Filion and Morin (1996). Depressions in the soil may favor higher seedling densities as germinants can benefit from runoff water pooling in the depressions. Furthermore, seeds can be washed down into and accumulate in depressions by heavy rains. However, the concave micro-topography of scarified plots can also reduce seedling survival by favoring excessive water accumulation and anaerobic conditions when there is poor drainage. We did not specifically assess seedling mortality resulting from flooding, predation or harvesting operations. Further investigation of the shelterwood and seed-tree systems in these ecosystems should take this into account (Frisque et al., 1978; Côté et al., 2003). Nevertheless, our findings indicate that seedling mortality a.c. was low overall, hence enabling the establishment of an abundant regeneration layer.

Previous research has shown that in black spruce stands the shelterwood system results in a significant growth response of residual trees and low mortality due to post-cutting windthrow (Montoro Girona et al., 2016, 2017). Here, we show that shelterwoods and ST harvesting followed by scarification result in an acceptable stocking and proportion of black spruce regeneration. Regeneration standards usually require that post-harvest stocking be equal to or greater than the stocking of the harvested stand (based on 4 m^2^ plots). In our study, the estimated stocking of the original stands ranged from 0.61–0.69 for the young and dense forests, and from 0.35 to 0.55 for the old and open ones. After 10 years, almost all treatments, including clear-cutting, resulted in a stocking equal or greater than 0.90 for both stand types. Hence, the shelterwood system followed by scarification did not increase the abundance of regeneration, compared to CC. However, the shelterwood treatments and scarification had a significant impact on the composition of the regeneration layer, as it increased the proportion of black spruce over balsam fir. The increased black spruce/balsam fir ratio is desirable, as it positively affects stand resilience to natural disturbances, such as fire and spruce budworm outbreaks, and preserves the economic value of future harvests.

Mini-strip shelterwood was the most efficient treatment for promoting black spruce regeneration. This harvesting variant had the highest proportion of trail surface per hectare and was the least expensive to implement due to the lack of tree selection. Our results demonstrate the importance of combining soil disturbance with partial cutting to create adequate seed-beds for black spruce. We expect that comparable results could be achieved at an even lower cost using disk trenching rather than spot scarification. Therefore, to evaluate these new silvicultural treatments in context of sustainable forest management objectives future research will be essential to determine the implantation costs and the biodiversity implications.

## Conclusions

Ensuring regeneration for adequate density and growth of conifers is one of the most challenging issues of boreal forest management. Our study provides a better understanding of the regeneration process in black spruce–dominated stands. We demonstrated that the experimental shelterwood and seed-tree systems followed by scarification are effective treatments for promoting regeneration in spruce-moss forest ecosystems. Black spruce regeneration was favored over balsam fir regeneration. The highest seedling densities were observed in the experimental shelterwood and seed-tree treatments. Soil disturbances were a key factor in the establishment success of black spruce, and insolation did not influence seedling density and growth, 10 years after cutting. Shelterwood and seed-tree systems followed by scarification enable an adequate regeneration in black spruce stands, confirming these treatments as viable silvicultural alternatives to clear-cutting when required by sustainable forest management objectives.

## Author contributions

MM and NT: conceptualization; MM: data curation and fieldwork; MM: formal analysis; MM, J-ML, NT, and HM: investigation; MM, J-ML, NT, and HM: methodology; MM: project administration; HM: resources; HM: supervision; MM, NT, and J-ML: validation; MM: visualization and edition; MM: writing–original draft; MM, NT, J-ML, and HM: writing–review: HM, J-ML, and MM: funding.

### Conflict of interest statement

The authors declare that the research was conducted in the absence of any commercial or financial relationships that could be construed as a potential conflict of interest.

## Supplementary material

Figure S1 Relationships between insolation and density of black spruce seedlings, 10 years after mini-strip shelterwood harvesting across a transect of plots, by stand type. PAR represents the percent transmittance of photosynthetically active radiation.

Figure S2 Seedling and sapling height-class distributions, 10 years after treatment by stand type and species; numbers from 1 to 4 represent each height class: (1) 0–4.9 cm; (2) 5–29.9 cm; (3) 30–99 cm; (4) >1 m.

Table S1 Initial stand characteristics by stand type for each study block. Data were obtained in permanent rectangular (10 × 60 m) sampling plots established in the center of the experimental units. Sampling covered the spatial heterogeneity of each silvicultural treatment (trails, edge and residual strip). Measurements were taken in 2002, one year before cutting (b.c.), on trees having a diameter at 1.3 m (DBH) of ≥9 cm for all tree species (*n* = 3,739). Stocking is the one expected from a random dispersion of trees, based on a Poisson distribution and the average tree density in 4 m^2^ plots.

Table S2 Stocking of main conifers species and hardwood regeneration, 10 years after cutting (mean ± standard error). Expected values assume the random distribution of seedlings and a density distribution following a Poisson distribution. Paper birch (*Betula papyrifera* Marsh) and aspen (*Populus tremuloides* Michx) were grouped as intolerant hardwoods.
