# Supplementary figures and images for: Conifer Regeneration After Experimental Shelterwood and Seed-Tree Treatments in Boreal Forests: Finding Silvicultural Alternatives
Source: Front Plant Sci. 2018 Aug 17;9:1145. doi: 10.3389/fpls.2018.01145 (PMC6108379; doi:10.3389/fpls.2018.01145)

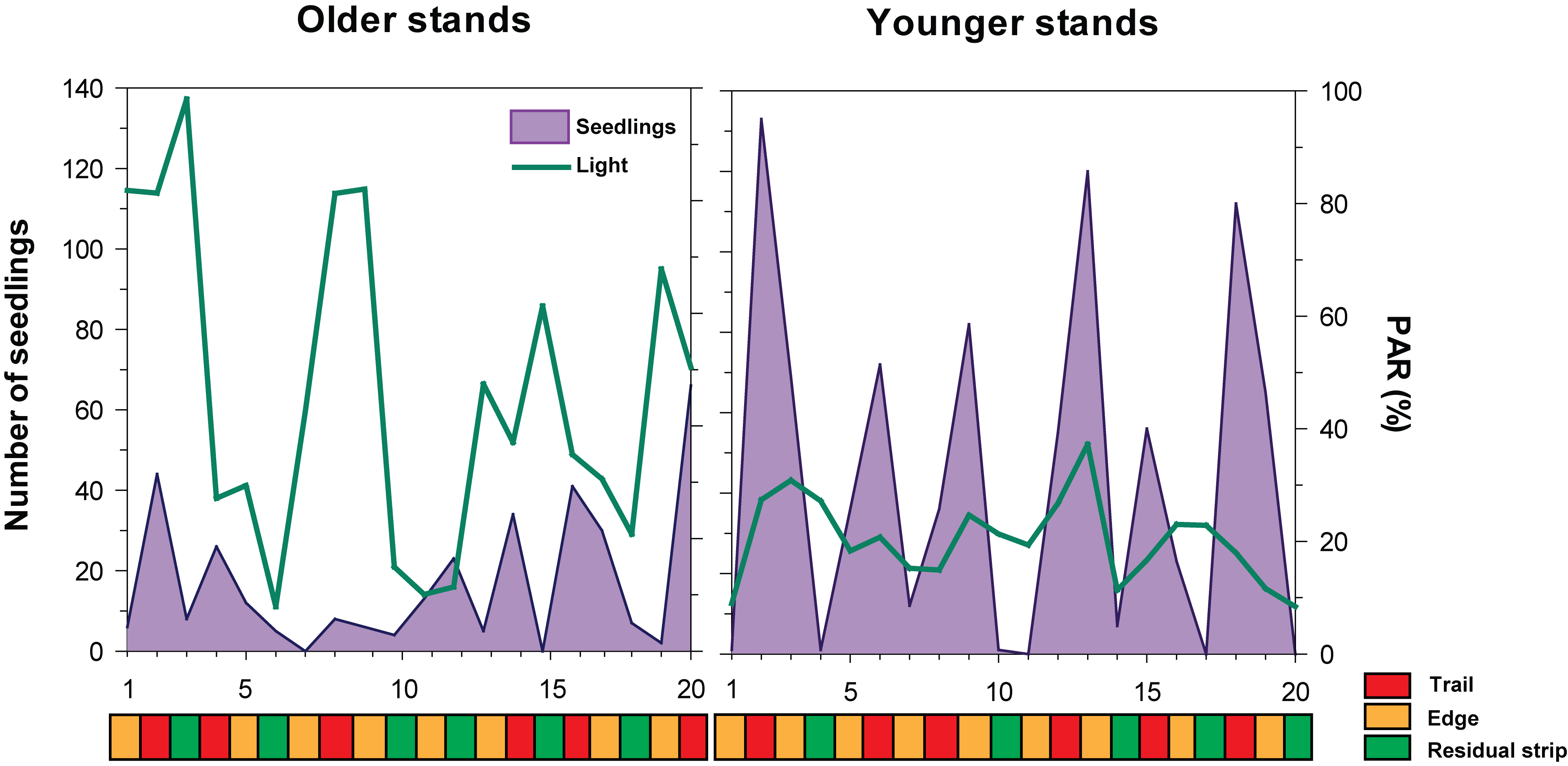

Supplement: Figure S1 — Relationships between insolation and density of black spruce seedlings, 10 years after mini-strip shelterwood harvesting across a transect of plots, by stand type. PAR represents the percent transmittance of photosynthetically active radiation. [file Image_1.TIF]

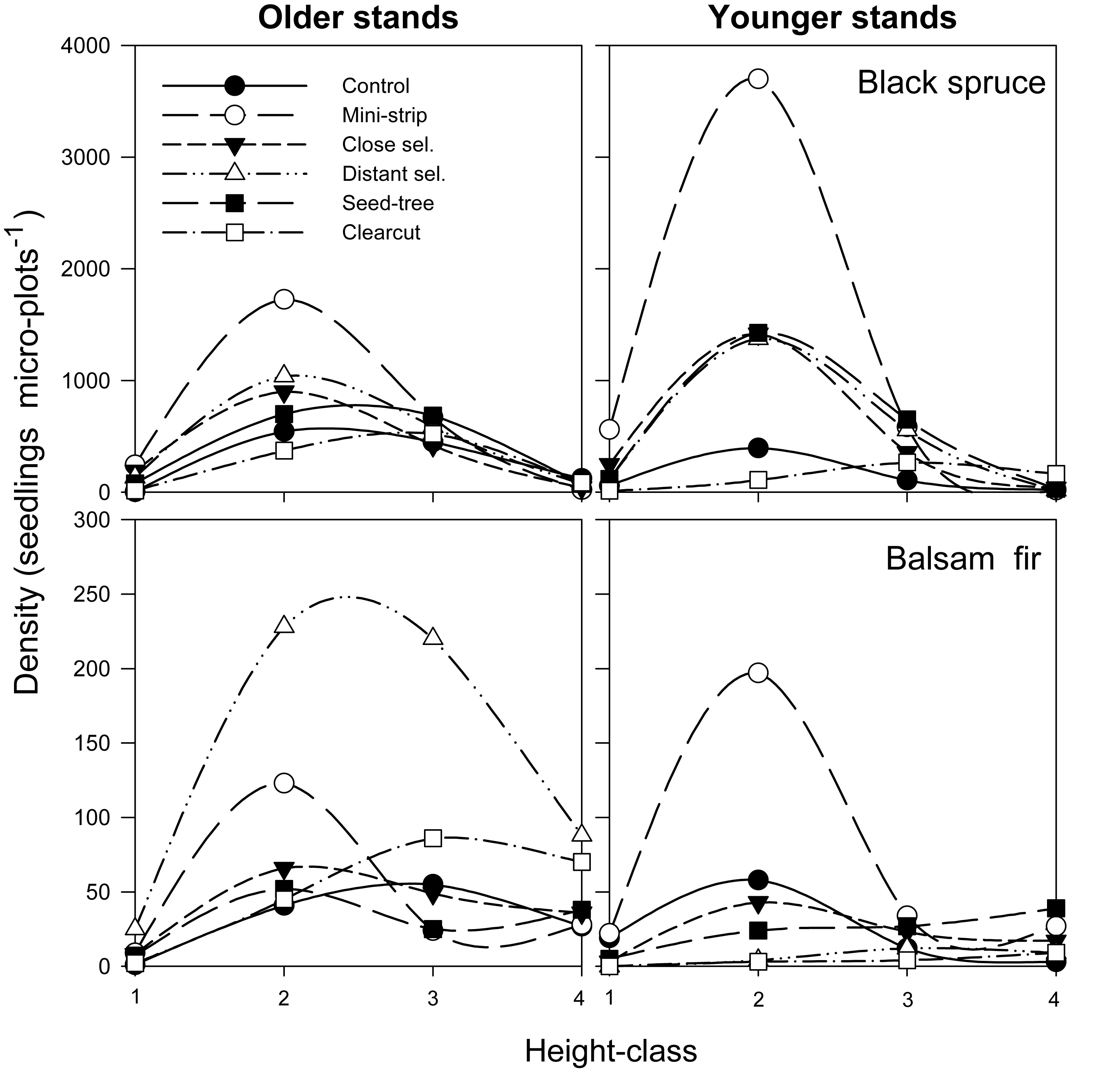

Supplement: Figure S2 — Seedling and sapling height-class distributions, 10 years after treatment by stand type and species; numbers from 1 to 4 represent each height class: (1) 0–4.9 cm; (2) 5–29.9 cm; (3) 30–99 cm; (4) >1 m. [file Image_2.TIF]
