# Supplementary material for: Conifer Regeneration After Experimental Shelterwood and Seed-Tree Treatments in Boreal Forests: Finding Silvicultural Alternatives
Source: Front Plant Sci. 2018 Aug 17;9:1145. doi: 10.3389/fpls.2018.01145 (PMC6108379; doi:10.3389/fpls.2018.01145)
Supplement: Table S1 — Initial stand characteristics by stand type for each study block. Data were obtained in permanent rectangular (10 × 60 m) sampling plots established in the center of the experimental units. Sampling covered the spatial heterogeneity of each silvicultural treatment (trails, edge and residual strip). Measurements were taken in 2002, one year before cutting (b.c.), on trees having a diameter at 1.3 m (DBH) of ≥9 cm for all tree species (n = 3,739). Stocking is the one expected from a random dispersion of trees, based on a Poisson distribution and the average tree density in 4 m2 plots. [file Table_1.pdf]

Table S1. Initial stand characteristics by stand type for each study block.

Stand type	Block	Density	Stocking	Dominant height	Diameter at breast height		Basal area	Volume	Species composition (%)			
					Mean	*Coefficient of variation*			*Black spruce*	*Balsam fir*	*Aspen*	*Paper birch*
		trees/ha		*m*	*cm*	*%*	*m²/ha*	*m³/ha*				
Younger	1	2542	0.64	17.0	15.0	22	45.7	248.6	97.0	1.3	0.2	1.1
	2	2325	0.61	17.3	14.4	24	39.5	209.2	94.6	3.5	1.6	0.3
	3	2919	0.69	17.4	12.8	31	41.9	251.3	95.7	0.0	4.2	0.1
Older	4	1981	0.55	17.1	15.3	22	37.7	209.0	98.8	0.7	0.3	0.2
	5	1556	0.46	18.1	16.4	24	34.2	202.6	97.0	2.5	0.3	0.2
	6	1075	0.35	18.3	15.7	28	22.8	134.9	92.5	7.5	0.0	0.0

Note: Data were obtained in permanent rectangular (10 × 60 m) sampling plots established in the centre of the experimental units. Sampling covered the spatial heterogeneity of each silvicultural treatment (trails, edge and residual strip). Measurements were taken in 2002, one year before cutting (b.c.), on trees having a diameter at 1.3 m (DBH)of ≥ 9 cm for all tree species (*n* = 3739). Stocking is the one expected from a random dispersion of trees, based on a Poisson distribution and the average tree density in 4 m^2 plots.
