# Supplementary material for: Conifer Regeneration After Experimental Shelterwood and Seed-Tree Treatments in Boreal Forests: Finding Silvicultural Alternatives
Source: Front Plant Sci. 2018 Aug 17;9:1145. doi: 10.3389/fpls.2018.01145 (PMC6108379; doi:10.3389/fpls.2018.01145)
Supplement: Table S2 — Stocking of main conifers species and hardwood regeneration, 10 years after cutting (mean ± standard error). Expected values assume the random distribution of seedlings and a density distribution following a Poisson distribution. Paper birch (Betula papyrifera Marsh) and aspen (Populus tremuloides Michx) were grouped as intolerant hardwoods. [file Table_2.pdf]

Table S2. Stocking of main conifers species and hardwood regeneration, 10 years after cutting (mean ± standard error). Expected values assume the random distribution of seedlings and a density distribution following a Poisson distribution. Paper birch (*Betula papyrifera* Marsh) and aspen (*Populus tremuloides* Michx) were grouped as intolerant hardwoods.

**Stand type**	**Period**	**Treatment**	**Stocking**							
			Black spruce		Balsam fir		Softwoods		Hardwoods	
			Measured	Expected	Measured	Expected	Measured	Expected	Measured	Expected
Older	Year 0		0.84(0.03)	1.00(0.00)	0.38(0.04)	0.94(0.02)	0.90(0.03)	1.00(0.00)	0.00(0.00)	0.00(0.00)
	Year 10	Control	0.81(0.04)	1.00(0.00)	0.21(0.04)	0.99(0.01)	0.90(0.03)	1.00(0.00)	0.01(0.01)	0.01(0.01)
		Mini-strip	0.94(0.02)	1.00(0.00)	0.36(0.04)	0.98(0.01)	0.96(0.02)	1.00(0.00)	0.53(0.04)	0.92(0.02)
		Close sel.	0.92(0.02)	1.00(0.00)	0.32(0.04)	0.98(0.01)	0.94(0.02)	1.00(0.00)	0.43(0.04)	0.82(0.03)
		Distant sel.	0.91(0.03)	1.00(0.00)	0.60(0.04)	1.00(0.00)	0.97(0.02)	1.00(0.00)	0.44(0.04)	0.93(0.02)
		Seed-tree	0.94(0.02)	1.00(0.00)	0.34(0.04)	0.95(0.02)	0.98(0.01)	1.00(0.00)	0.56(0.04)	0.96(0.02)
		Clear-cut	0.83(0.03)	1.00(0.00)	0.32(0.04)	0.99(0.01)	0.87(0.03)	1.00(0.00)	0.71(0.04)	1.00(0.00)
Younger	Year 0		0.52(0.04)	0.98(0.01)	0.21(0.04)	0.42(0.04)	0.61(0.04)	0.90(0.03)	0.03(0.01)	0.03(0.01)
	Year 10	Control	0.49(0.04)	1.00(0.00)	0.31(0.04)	0.91(0.03)	0.62(0.04)	1.00(0.01)	0.08(0.02)	0.08(0.02)
		Mini-strip	0.88(0.03)	1.00(0.00)	0.56(0.04)	0.98(0.01)	0.93(0.02)	1.00(0.00)	0.56(0.04)	0.99(0.01)
		Close sel.	0.90(0.03)	1.00(0.00)	0.29(0.04)	0.90(0.03)	0.90(0.03)	1.00(0.00)	0.61(0.04)	0.99(0.01)
		Distant sel.	0.71(0.04)	1.00(0.00)	0.14(0.03)	0.76(0.04)	0.74(0.04)	1.00(0.00)	0.43(0.04)	0.93(0.02)
		Seed-tree	0.88(0.03)	1.00(0.00)	0.29(0.04)	0.93(0.02)	0.90(0.03)	1.00(0.00)	0.69(0.04)	1.00(0.00)
		Clear-cut	0.87(0.03)	1.00(0.00)	0.09(0.03)	0.76(0.04)	0.87(0.03)	0.99(0.01)	0.60(0.04)	0.98(0.01)
